# Chained multimediator model of sexual orientation disclosure, sexual minority stigma, sexual minority identity, social support, and resilience among ymsms

**DOI:** 10.1186/s12889-022-13231-8

**Published:** 2022-04-21

**Authors:** Sumin Tan, Ping Cen, Ting Fang, Xing Yang, Yun Zhang, Jiawen Zhu, Haimei Huang, Min Wang, Li Jiang, Jieling Mo, Chuanyi Ning

**Affiliations:** 1 Nursing College, Guangxi Medical University, Nanning, Guangxi China; 2 Nanning Municipal Center for Disease Prevention and Control, Nanning, Guangxi China; 3 School of Public Health, Guangxi Medical University, Nanning, Guangxi China

**Keywords:** Sexual minority stigma, Sexual minority identity, Social support, Resilience, Sexual orientation disclosure, Mediation model, YMSM

## Abstract

**Introduction:**

This study aimed to investigate sexual orientation disclosure and mental health among young men who have sex with men (YMSMs). To this end, we constructed a chained multimediator model of sexual minority stigma, sexual minority identity, social support, and resilience, with the moderator of sexual orientation disclosure.

**Methods:**

We conducted a cross-sectional survey of 345 YMSMs in Nanning, China. Bivariate analysis was used to evaluate factors associated with sexual orientation disclosure. Sexual minority stigma was used to predict identity, with social support as the step 1 mediator and resilience as the step 2 mediator. Sexual minority identity was analyzed using a chained moderated mediation model; sexual orientation disclosure was included as a moderator in all models to control its confounding effect.

**Results:**

The average age of YMSMs was 20.0 ± 1.3 years. Bivariate analysis indicated that YMSMs who disclosed sexual orientation may have experienced less stigma (15.49 ± 3.02 vs 16.21 ± 2.74), obtained more social support (65.98 ± 11.18 vs 63.19 ± 11.13), had strong psychological resilience (37.40 ± 8.57 vs 35.39 ± 7.73), and had a more positive self-identity (104.12 ± 21.10 vs 95.35 ± 16.67); differences between subgroups were statistically significant (*p* < 0.05). Sexual minority stigma, perceived stigma, and enacted stigma were significantly associated with social support and resilience. The association between sexual minority stigma and sexual minority identity was significantly mediated by social support (indirect effect [95% CI] =  − 3.307 [− 4.782, − 1.907]). Resilience significantly mediated the same association for identity (− 2.544 [− 4.052, − 1.114]). The chained relationship from sexual minority stigma to social support, resilience, and identity was also significant, with an indirect effect of − 0.404 [− 0.621, − 0.249].

**Conclusion:**

Among YMSMs in China, sexual minority stigma affects sexual minority identity through social support and resilience. Given the psychological effects of stigma, social support and resilience must be considered to better promote positive self-identity and mental health among YMSMs.

## Introduction

It has been estimated that 83.0% of the global sexual minority population conceals its sexual orientation from all or most people [1]. Such concealment may cause men who have sex with men (MSM) to avoid HIV testing, thereby increasing the risk of HIV infection and interfering with AIDS-related behavioral interventions [2–4]. Although the experience of sexual stigma among MSMs in China is well documented [5–8], little is known about its effect on the psychological well-being of young MSMs (YMSM). YMSMs (aged 15–24) who perceive or experience stigma are in a period of changing and unstable physiology and psychology, and they are therefore more sensitive to stigma and negative evaluations [9–12]. Yet, few studies have specifically investigated potential moderating and mediating effects in the relationship between sexual stigma and psychological distress among YMSMs in China.

*Sexual minority stigma* refers to the social and structural devaluation of lesbian, gay, bisexual, and other sexually diverse people and the associated power inequalities, negative attitudes, and stereotypes [13]. “Coming out” can still pose a huge dilemma for sexual minorities in many countries, even with same-sex marriage becoming increasingly common [14–16]. The low rate of sexual orientation disclosure can also affect sexual behaviors (e.g., getting tested for sexually transmitted infections) and mental health [17–20]. Traditional concepts of marriage and childbirth are deeply rooted in China, and sexual minority stress is therefore high among Chinese MSMs [21, 22]. This heteronormative social environment [23] results in severe marginalization and stigma for MSMs [24], who may be exposed to negative experiences, such as social rejection, isolation, diminished social support, discrimination, and verbal and physical abuse [25]. The resulting negative effects for MSMs can include depression, anxiety, tension, and fear, as well as violence and a propensity for suicidal and antisocial behavior [26–29].

*Sexual minority identity* refers to one’s sense of belonging to a sexual minority [30]. Previous studies have described identity formation and integration as a process in which individuals strive for congruence in their sexual orientation in areas such as sexual attraction, thought, and fantasy [31–35]. Sexual minority individuals are often raised in communities that are ignorant of or openly hostile toward homosexuality and therefore may have difficulty forming a positive identity [26, 36]. The development of sexual identity is a difficult, complex, multidimensional process [37]. As an important factor of MSMs’ mental health, among different types of sexual minority, the effect of sexual minority identity on risky behavior is different and enhances the rise [38–40]. While “coming out” is typically stressful for YMSMs, it is also associated with positive mental health and identity outcomes, especially in the long run [41].

MSM research has consistently shown that parental and peer support are related to good mental health (e.g., high self-esteem, less depression, reduced suicidality), self-acceptance, and overall well-being [12, 42–44]. *Resilience* is the ability to have good psychological outcomes and quality of life despite experiencing stressful environments or other serious adversities [45, 46]. People with high resilience have reported a lower prevalence of psychological distress or disorders [47, 48].

In light of the above, social support and resilience might be considered to mediate the effect of sexual minority stigma perception on identity. This might further suggest a potential chained mechanism by which social support and resilience mediate the relationship between sexual minority stigma and sexual minority identity. In addition to the direct effect, stigma may exert indirect effects on identity by enhancing social support and resilience. To our knowledge, no previous study has investigated this potential chained mediation mechanism.

This study investigated the relationship between mental health and sexual orientation disclosure. It also examined the complex underlying mechanisms linking sexual minority stigma to identity through two chained mediators: social support and resilience. To this end, we analyzed data collected from a probability sample of YMSMs in China. The findings can enhance our understanding of the mechanisms of sexual minority identity and provide a reference for interventions aiming to increase the acceptance and positivity of sexual identity.

## Methods

### Participants

Participants were recruited from July 2019 to July 2020 with support from the Voluntary Counseling and Testing (VCT) clinic of the Centers for Disease Control and Prevention (CDC), Guangxi, China. Participants were also recruited from nongovernmental organizations (NGOs) (e.g., Rainbow of Green City) in Nanning China. We targeted YMSMs who were aged 18–24, who self-reported receptive or insertive anal intercourse or oral sex with another man in the last six months, who had not previously tested positive for HIV, and who agreed to participate in the study.

### Procedure

Each survey site was assigned to two well-trained researchers, who were responsible for recruiting participants and distributing the survey. It was an anonymous self-reported questionnaire survey. After providing informed consent, participants received free HIV testing. We collected participants’ fingertip blood, which was placed on HIV testing reagents. As they waited for the HIV testing results, participants were asked to complete the questionnaires. They filled out the questionnaires independently in a separate room to protect their privacy. In-person assistance was available if participants had any questions about the survey. Most took ~ 30 min to complete the questionnaire. Participants received 50 RMB (approximately USD 8) after completing the questionnaire. Among 350 eligible YMSMs, 345 were retained for analysis after excluding individuals with incomplete data for key variables (completion rate: 98.6%).

### Measurement

#### Demographics

The demographic variables included age, ethnicity, education, employment status, marital status, monthly income, and sexual orientation. Ethnicity was Han, Zhuang, or other minority. Education was high school or below or college or above. Identity was student, employee, farmworker, or unemployed. Marital status was unmarried or married/divorced. Monthly income (in RMB) was ≤ 3000, 3001–5000, or > 5000. Sexual orientation was gay, bisexual, or undecided. For the descriptive analysis, we separated the sociodemographic and measurement scale information according to whether participants had disclosed their sexual orientation.

### Mediation model

**Fig. 1:**
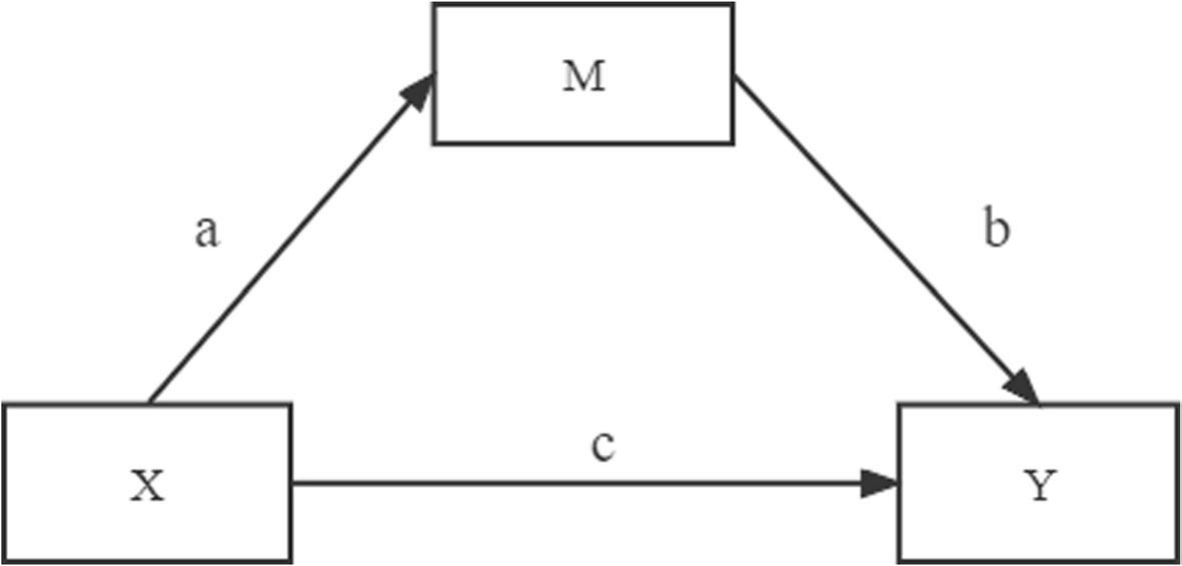
Simple mediator model

Mediation model is assumed that there is a causal influence between independent variable X and dependent variable Y, and this influence is realized with the participation of the third variable M. In other words, the influence of independent variable X on dependent variable Y is partly indirect through the intermediate variable M, which is called M as the mediator variable (Fig. 1) [49]. A model that has multiple mediators explaining the effect of an independent variable on a dependent variable is called multiple mediation. compared to a direct effect on the regression analysis and other methods, mediation analysis can further explain the mechanism behind the causal variables.

### Predictor

#### Sexual minority stigma

Sexual minority stigma was evaluated using Neilands’s questionnaire, Assessment of Stigma Toward Homosexuality in China [50]. This scale has been used to measure stigma against the MSM population in China and the US and has good reliability and validity (Cronbach’s alpha: 0.75). We measured two subscales of YMSMs’ sexual minority stigma: perceived stigma and enacted stigma. Items for perceived stigma included “How often have you heard that homosexuals are not normal?”; “How often have you felt that your homosexuality hurt and embarrassed your family?”; and “How often have you had to pretend that you are not homosexual in order to be accepted?” Enacted stigma refers to overt experiences of discrimination, including physical, verbal, and sexual violence and hate crimes. Items included “You’ve been hit, beaten, physically attacked, or sexually assaulted”; “You’ve been fired from your job or denied a job or promotion”; and “You’ve been prevented from moving into a house or apartment by a landlord or realtor.” Each item had four response options: 1 = never, 2 = once or twice, 3 = a few times, and 4 = many times. Mean stigma scores were computed for total stigma and two subscales, such that higher scores reflected more stigma experiences.

### Mediator

#### Social support

Social support was measured using the Multidimensional Perceived Social Support Scale [51]. It includes 12 entries divided into three dimensions: support from families, friends, and others. Sample items included “I can get emotional help and support from my family when I need it,” and “I can rely on my friends in times of trouble.” Response options ranged from 1 (very strongly disagree) to 7 (very strongly agree). The higher the overall score and the higher the score in each dimension, the more social support the individual perceived. The Cronbach’s α coefficient of the scale was 0.96.

### Mediator

#### Resilience

A modified Connor–Davidson Resilience Scale [52] was used to measure psychological resilience. Sample items included “I bounce back after illness or injury” and “Under pressure, I stay focused.” These were divided into three dimensions: target focus, emotional control, and positive cognitive. Responses were given on a five-point scale ranging from 0 (“not at all”) to 4 (“extremely”). A higher total score represented a higher level of resilience. The scale exhibited good internal reliability (Cronbach’s alpha: 0.96).

### Moderator

#### Sexual orientation disclosure

*Sexual orientation disclosure* was defined as having ever disclosed one’s sexual orientation to anyone other than a sexual partner. Healthcare professional disclosure means disclosing to a doctor or other medical provider. Studies have indicated that the association between sexual minority stigma and sexual minority identity differs by orientation disclosure [25, 53]. To better assess the proposed chained mediation mechanism, this variable was used as a moderator and was assessed as whether sexual orientation was disclosed (yes/no).

### Outcome

#### Sexual minority identity

Sexual minority identity was measured using the Lesbian, Gay, & Bisexual Identity Scale [37]. It is divided into eight dimensions: acceptance of attention (three items), hidden motivation (three items), identity hesitation (four items), internalization homogeneity (three items), difficult process (three items), identity advantage (three items), identity verification (three items), and identity center (five items) (total items: 27). Responses included strongly agree (six points), agree (five points), relatively agree (four points), relatively disagree (three points), disagree (two points), and strongly disagree (one point). The eleventh and twenty-third items were reverse scored. The higher the score, the higher the degree of negative identity. Cronbach’s alpha was 0.72.

### Statistical analysis

**Fig. 2:**
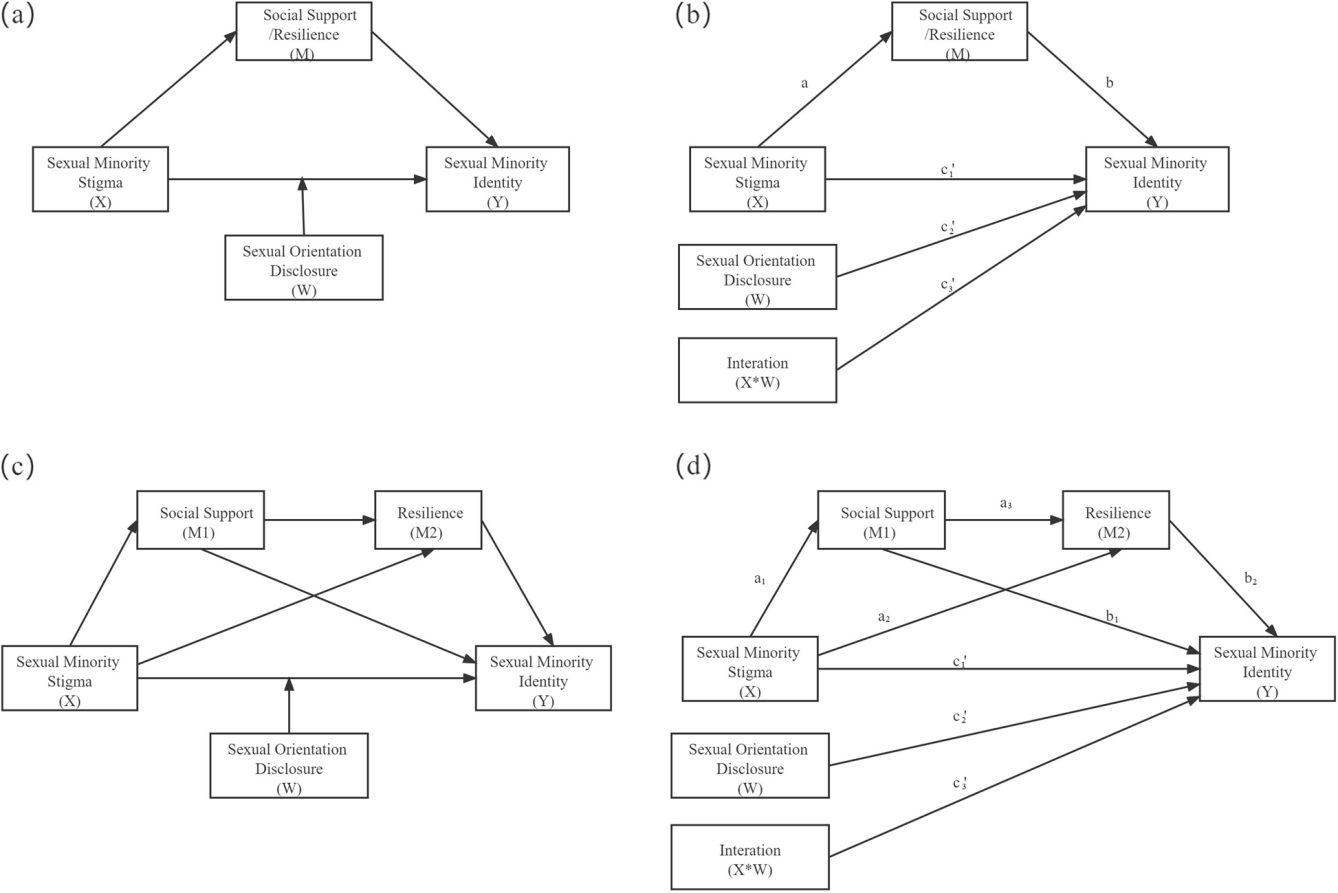
Conceptual (**a**, **c**) and statistical (**b**, **d**) illustrations of mediation modeling (upper panel) and chained mediation modeling (bottom panel). Note: X = predictor; M, M1, and M2 = mediators; Y = outcome; and W = moderator. Orientation disclosure was modeled as the moderator

Descriptive analyses were used to describe the study sample and for bivariate analysis by the sexual orientation disclosure subgroup. We tested the proposed chained mediation model in three steps. In step 1, Pearson’s correlation was used to investigate correlations among the key variables, including sexual minority stigma, social support, resilience, and sexual minority identity. In step 2, moderated mediation modeling was used to test the individual roles of social support and resilience in mediating the association between sexual minority stigma and sexual minority identity (i.e., sexual minority stigma → social support → sexual minority identity, and sexual minority stigma → resilience → sexual minority identity), considering the moderating role of orientation disclosure. As shown in Fig. 2a and 2b, the product of the estimated coefficients a and b (a*b) provided a measure of the indirect effect of sexual minority stigma on identity through social support/resilience. A significant c_3_’ provided a measure of the moderating effect of orientation disclosure. In step 3, chained mediation modeling with two mediators was conducted. As shown in Fig. 2c and d, the products of the estimated coefficients a_1_ and b_1_ (a_1_*b_1_), a_2_ and b_2_ (a_2_*b_2_), and a_1_, a_3_, and b_2_ (a_1_*a_3_*b_2_) provided measures of the indirect effects of sexual minority stigma → social support → sexual minority identity, sexual minority stigma → resilience → sexual minority identity and sexual minority stigma → social support → resilience → sexual minority identity, respectively.

Two investigators used Epidata 3.1 to enter the questionnaire data, save the final database after consistency checks, and import it into SPSS 24.0 for descriptive analysis. Type I error was set at *p* < 0.05 for statistical inference. Moderated mediation analyses were conducted using Mplus 8.3.

## Results

### Study sample characteristics

A total of 61.7% of participants were Han, and one-third were Zhuang; 79.1% had a college education or higher; 50.1% were staff; 56.2% made 3000 RMB or more; 67.8% reported their sexual orientation as gay. The chi-squared test showed significant statistical differences in sexual orientation disclosure between the self-reported sexual orientation groups (Table 1).

### Bivariate analysis by sexual orientation disclosure subgroup

In the bivariate analysis *t*-test, there were statistical differences in the scores for sexual minority stigma, social support, resilience, and sexual minority identity between subgroup according to whether sexual orientation was disclosed. YMSMs who disclose sexual orientation might experience less stigma, obtain more social support, have strong psychological resilience, and have a positive self-identity. However, different from perceived stigma, enacted stigma showed no significant statistical difference between subgroups (Table 2). For sexual minority stigma, the subgroup of YMSMs who disclosed sexual orientation had less perceived stigma. For the social support aspect, family support did not play any special role in the effect; YMSMs received more support from friends and others, depending on sexual orientation disclosure. Each dimension of resilience was significantly different between the two subgroups; high scorers tended to be “out.” For sexual minority identity, except for the dimension of internalized homonegativity, identity superiority and identity affirmation played special roles in the effect. Other dimensions showed that with higher scores, YMSMs with a more positive sexual minority identity tended to disclose their sexual orientation.

**Table 1:** Sample characteristics by sexual orientation disclosure subgroup (*N*=345)

Variables	N (%)	Sexual Orientation Disclosure			
		Yes = 188	No = 157	χ^2^	*p*
		[*n* (%)]	[*n* (%)]		
Nationality				1.145	0.564
Han	213 (61.7)	115 (61.2)	98 (62.4)		
Zhuang	124 (35.9)	70 (37.2)	54 (34.4)		
Other minority	8 (2.3)	3 (1.6)	5 (3.2)		
Education				0.108	0.743
High school or below	72 (20.9)	38 (20.2)	34 (21.7)		
College or above	273 (79.1)	150 (79.8)	123 (78.3)		
Employment status				3.059	0.383
Student	114 (33.0)	69 (36.7)	45 (28.7)		
Employee	173 (50.1)	89 (47.3)	84 (53.5)		
Farmworker	18 (5.2)	8 (4.3)	10 (6.4)		
Unemployed	40 (11.6)	22 (11.7)	18 (11.5)		
Marital status				0.058	0.809
Unmarried	338 (98.0)	185 (98.4)	153 (97.5)		
Married/divorced	7 (2.0)	3 (1.6)	4 (2.5)		
Monthly income				3.535	0.171
≤ 3000	151 (43.8)	87 (46.3)	64 (40.8)		
3001–5000	95 (27.5)	44 (23.4)	51 (32.5)		
> 5000	99 (28.7)	57 (30.3)	42 (26.8)		
Sexual orientation				16.390	0.000
Gay	234 (67.8)	145 (77.1)	89 (56.7)		
Bisexual	96 (27.8)	37 (19.7)	59 (37.6)		
Undecided	15 (4.3)	6 (3.2)	9 (5.7)		
HIV testing frequency				3.335	0.189
About 3 months/time	155 (43.5)	88 (46.8)	62 (39.5)		
About 6 months/time	55 (15.9)	32 (17.0)	23 (14.6)		
About 12 months/time	140 (40.6)	68 (36.2)	72 (45.9)		

### Correlations among predictors, mediators, moderators, and outcomes

Sexual minority stigma, perceived stigma, and enacted stigma were significantly associated with social support and resilience. Social support was significantly associated with resilience; both were significantly associated with sexual minority identity. This supports the proposed mediation models. Additionally, Table 3 reveals differences in the correlations according to whether participants disclosed sexual orientation, suggesting a need to control this variable as a moderator (Table 3). YMSMs who disclosed sexual orientation reported less perceived stigma but more enacted stigma compared to those who concealed sexual orientation. YMSMs who did not disclose sexual orientation might have experienced less enacted stigma; there was no significant association with sexual minority identity.

**Table 2:** Scores for measurement scales and dimensions by sexual orientation disclosure subgroup (*N* = 345)

Variables	Sexual Orientation Disclosure Yes = 188 No = 157		*t*	*p*
Sexual minority stigma	15.49 ± 3.02	16.21 ± 2.74	-2.301	0.022
Perceived stigma	7.95 ± 2.34	8.72 ± 2.36	-3.021	0.003
Enacted stigma	7.54 ± 1.55	7.50 ± 1.50	0.245	0.806
Social support	65.98 ± 11.18	63.19 ± 11.13	2.312	0.021
Family support	20.37 ± 4.402	19.60 ± 4.259	1.636	0.103
Friend support	22.73 ± 3.970	21.73 ± 3.938	2.336	0.020
Significant other	22.88 ± 3.834	21.86 ± 3.788	2.481	0.014
Resilience	37.40 ± 8.57	35.39 ± 7.73	2.265	0.024
Target focus	13.51 ± 5.052	12.10 ± 5.393	2.501	0.013
Emotional control	10.59 ± 4.125	9.42 ± 4.304	2.572	0.011
Positive cognition	10.58 ± 4.007	9.45 ± 4.270	2.526	0.012
Sexual minority identity	104.12 ± 21.10	95.35 ± 16.67	4.211	0.000
Acceptance concerns	10.46 ± 4.471	8.75 ± 3.846	3.823	0.000
Concealment motivation	6.09 ± 2.722	4.62 ± 2.067	5.680	0.000
Identity uncertainty	18.21 ± 4.438	16.18 ± 4.384	4.239	0.000
Internalized homonegativity	11.25 ± 3.580	10.62 ± 3.491	1.635	0.103
Difficult process	11.03 ± 2.875	10.00 ± 2.837	3.340	0.001
Identity superiority	14.68 ± 3.176	14.26 ± 2.656	1.337	0.182
Identity affirmation	12.06 ± 4.753	11.76 ± 4.125	0.640	0.523
Identity centrality	20.34 ± 6.087	19.15 ± 5.275	1.932	0.054

### Moderated mediation modeling of sexual minority identity

We analyzed data of moderated mediation modeling in Fig. 2b, Social support significantly mediated the association between total sexual minority stigma and sexual minority identity (sexual minority stigma → social support: − 1.027 [− 1.400, − 0.683]; social support → sexual minority identity: 0.597 [0.459, 0.740]; indirect effect =  − 0.613 [− 0.886, − 0.394]). Resilience significantly mediated the association between total sexual minority stigma and identity (indirect effect =  − 0.790 [− 1.128, − 0.518]). Further analyses showed that resilience mediated the association between perceived stigma and enacted stigma; more details can be found in Table 4. YMSMs with less social support experienced higher sexual minority stigma and were more likely to have a negative sexual minority identity. Additionally, resilience had a positive mediating effect on the path connections of sexual minority stigma and sexual minority identity among YMSMs. Individuals with higher stigma had less resilience, which in turn reduced positive identity.

**Table 3:** Correlations between sexual minority stigma, social support, resilience, and sexual minority identity among YMSMs

Variables	Mean (SD)	2	3	4	5	6
**Sexual Orientation Disclosure = Yes (***N*** = 188)**						
1. Sexual minority stigma	15.49 (3.02)	0.86^**^	0.65^**^	− 0.25^**^	− 0.26^**^	− 0.40^**^
2. Perceived stigma	7.95 (2.34)		0.17^*^	− 0.24^**^	− 0.20^**^	− 0.40^**^
3. Enacted stigma	7.54 (1.55)			− 0.14^*^	− 0.20^*^	− 0.18^*^
4. Social support	65.98 (11.18)				0.56^**^	0.51^**^
5. Resilience	37.40 (8.57)					0.67^**^
6. Sexual minority identity	104.12 (21.10)					
**Sexual Orientation Disclosure = No (***N* = 157)						
1. Sexual minority stigma	16.21 (2.74)	0.84^**^	0.51^**^	− 0.26^**^	− 0.15^*^	− 0.29^**^
2. Perceived stigma	8.72 (2.36)		0.04	− 0.21^**^	− 0.07^**^	− 0.26^**^
3. Enacted stigma	7.50 (1.50)			− 0.14^*^	− 0.16^*^	− 0.13
4. Social support	63.19 (11.13)				0.45^**^	0.28^**^
5. Resilience	35.39 (7.73)					0.43^**^
6. Sexual minority identity	95.35 (16.67)					

Note: **p < 0.001, *p < 0.05. Weight was considered when estimating correlations

**Table 4:** Moderated mediation model of associations between sexual minority stigma and sexual minority identity among YMSMs

Variables	Coefficients	95% confidence intervals
Moderator W: Sexual orientation Disclosure		
**Predictor X: Sexual minority stigma**		
**Mediator M**_**1**_**: Social support**		
X → M_1_ (a)	− 1.027	− 1.400, − 0.683
M_1_ → Y with X (b)	0.597	0.459, 0.740
X → Y with M_1_ (c_1_’)	− 3.307	− 4.782, − 1.907
W → Y (c_2_’)	− 22.861	− 38.34, − 7.518
X*W → Y (c_3_’)	1.072	− 0.146, 2.014
Indirect effect (a* b)	− 0.613	− 0.886, − 0.394
**Mediator M**_**2**_**: Resilience**		
X → M_2_ (a)	− 0.644	− 0.877, − 0.432
M_2_ → Y with X (b)	1.227	1.059, 1.396
X → Y with M_2_ (c_1_’)	− 2.544	− 4.052, − 1.114
W → Y (c_2_’)	− 15.394	− 29.779, − 0.588
X*W → Y (c_3_’)	0.645	− 0.272, 1.543
Indirect effect (a*b)	− 0.790	− 1.128, − 0.518
**Predictor X: Perceived stigma**		
**Mediator M**_**1**_**: Social support**		
X → M_1_ (a)	− 1.139	− 1.504, − 0.778
M_1_ → Y with X (b)	0.090	− 0.075, 0.256
X → Y with M_1_ (c_1_’)	− 1.569	− 3.773, 0.682
W → Y (c_2_’)	− 9.466	− 22.300, 3.106
X*W → Y (c_3_’)	1.005	− 0.446, 2.458
Indirect effect (a*b)	− 0.103	− 0.315, 0.081
**Mediator M**_**2**_**: Resilience**		
X → M_2_ (a)	− 0.560	− 0.840, − 0.274
M_2_ → Y with X (b)	0.222	0.000, 0.437
X → Y with M_2_ (c_1_’)	− 1.416	− 3.650, 0.830
W → Y (c_2_’)	− 8.502	− 21.619, 3.914
X*W → Y (c_3_’)	0.911	− 0.522, 2.383
Indirect effect (a*b)	− 0.125	− 0.308, − 0.014
**Predictor X: Enacted stigma**		
**Mediator M**_**1**_**: Social support**		
X → M_1_ (a)	− 1.008	− 2.225, − 0.318
M_1_ → Y with X (b)	0.089	− 0.081, 0.253
X → Y with M_1_ (c_1_’)	0.627	− 2.103, 3.942
W → Y (c_2_’)	4.034	− 12.195, 19.165
X*W → Y (c_3_’)	− 0.691	− 2.634, 1.356
Indirect effect (a* b)	− 0.090	− 0.408, 0.061
**Mediator M**_**2**_**: Resilience**		
X → M_2_ (a)	− 0.992	− 1.571, − 0.659
M_2_ → Y with X (b)	0.229	0.003, 0.446
X → Y with M_2_ (c_1_’)	− 0.865	− 1.853, 4.098
W → Y (c_2_’)	4.772	− 11.202, 19.742
X*W → Y (c_3_’)	− 0.760	− 2.612, 1.307
Indirect effect (a*b)	− 0.227	− 0.532, − 0.005

### Chained moderated mediation modeling of sexual minority identity

**Fig. 3:**
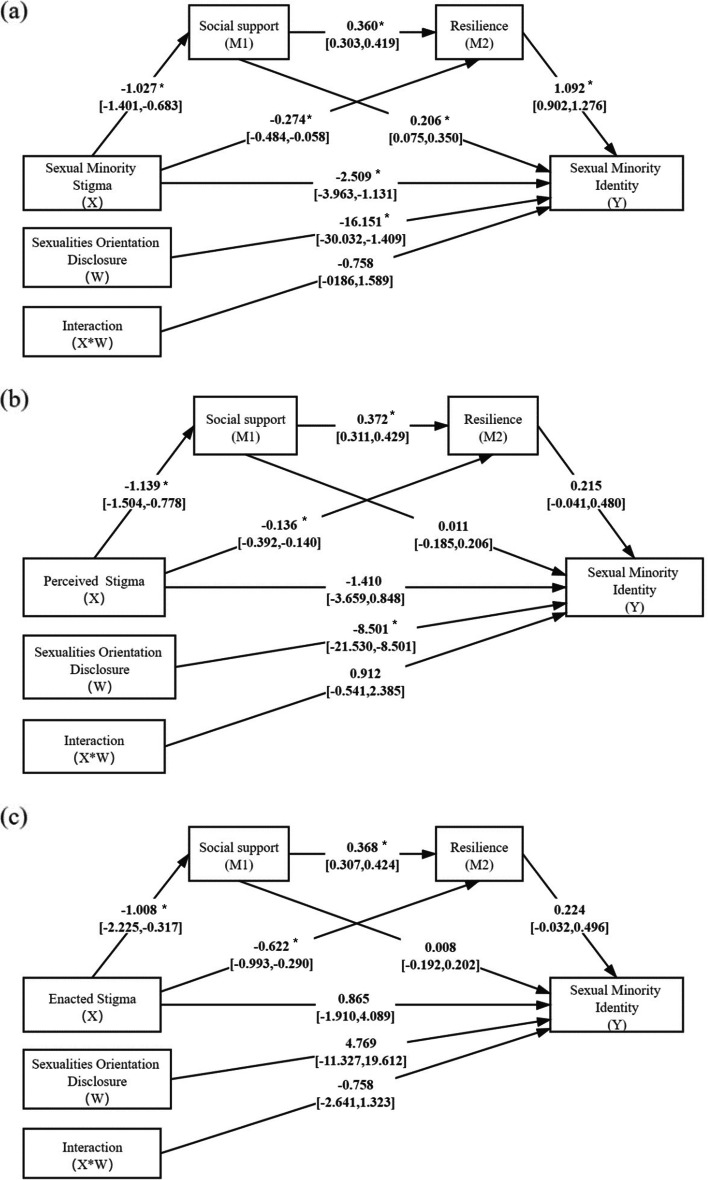
Chained mediation modeling of the associations among sexual minority stigma, social support, resilience, and sexual minority identity. Sexual orientation disclosure was modeled as the moderator; nationality gender, education, and marital status were included as covariates. **a** X → M1 → M2 → Y: − 0.404 [− 0.621, − 0.249]; **b** X → M1 → M2 → Y: -0.091 [− 0.231, 0.010]; **c** X → M1 → M2 → Y: 0.083 [− 0.296, 0.002] *: *p* < 0.05, statistically significant

The results shown in Fig. 3a indicate that total sexual minority stigma was significantly associated with social support (coefficient [95% CI] =  − 1.027 [− 1.401, − 0.683]). This in turn was associated with resilience (coefficient = 0.360 [0.303, 0.419]) and further associated with sexual minority identity (coefficient = 1.092 [0.902, 1.276]). The chained two-step indirect effect of sexual minority stigma → social support → resilience → sexual minority identity was − 0.404 [− 0.621, − 0.249]. The results in Fig. 3b show that social support and resilience did not significantly mediate the association between perceived stigma and sexual minority identity. The indirect effect of perceived stigma → social support → resilience → sexual minority identity was − 0.091 [− 0.231, 0.010]. The results in Fig. 3c show the mediating role of employment uncertainty and anxiety between enacted stigma and sexual minority identity. The indirect effect for the path enacted stigma → social support → resilience → sexual minority identity was − 0.083 [− 0.296, 0.002]. In these moderated mediation models, social support was not directly associated with sexual minority identity (0.111 [− 0.185, 0.206] for perceived stigma; 0.008 [-0.192, 0.202] for enacted stigma). Further, resilience was not directly associated with sexual minority identity (0.215 [− 0.041, 0.480] for perceived stigma; 0.224 [− 0.032, 0.496] for enacted stigma).

## Discussions

As the concept opens up, we can find the number of YMSMs disclosure sexual orientation has increased, about half of YMSM have told others about self-sexual orientation. Our results revealed a significant relationship between mental health and sexual orientation disclosure among Chinese YMSMs. YMSMs who were “coming out” were more likely to have good mental health, as in previous studies [19]. So, we can adopt more ways to advocate society and YMSMs for greater acceptance of homosexual orientation, education, and promotion of positive values and equality may help decrease psychological distress and develop a healthy mindset for YMSMs [54, 55].

One important finding is the demonstration of the effect mechanism of stigma on sexual minority identity, mediated by social support and resilience. Social support and resilience are two important factors related to self-identity [54, 56]. High levels of resilience better reduce the impact of stigma, for example, counseling or individual psychotherapy could alleviate negative effects on self-perception [57]. Our results indicated support from friends or peer are more effective impact discrimination perception than family support and work colleagues’ support, so peer care and help service is necessary. And stigma is a multifaceted concept [26, 58–60]. There are different effects in the mediation model. *Perceived stigma* refers to expectations of stigma and prejudice, which cause stress by requiring vigilance. It is assessed as a person’s level of awareness of being stigmatized and devalued by their community [61]. *Enacted stigma* refers to experiences of victimization, harassment, threats, and discrimination in daily life, at work, and in housing situations [62]. For these differences, corresponding interventions require our future research.

Our findings also revealed a chained mediation mechanism in which the relationship between sexual minority stigma and identity was mediated by both social support and resilience. Other studies have also observed indirect effects through the associated mediators of social support and resilience [54, 57, 63, 64]. This chained mediation mechanism highlights the importance of a no-stigma environment and social support for YMSMs in China. Social support from family, friends, communities, and medical institutions can mitigate the effects of stigma and positively affect self-identity. Social support also plays an important role by providing informational, instrumental, and emotional support. Resilience increases self-worth and reduces stress, thus helping YMSMs to have a more positive sexual minority identity and increasing their receptiveness to HIV risk-reduction counseling [65, 66].

A positive sexual minority identity among YMSMs can reduce the effects of perceived stigma; it has chain mediating effects in the two key target variables of social support and resilience. Compared to total stigma, the effects of perceived stigma and enacted stigma were not significant; this warrants additional research in the future.

This study has some limitations. First, it was a cross-sectional study limited to one city in China; caution should be exercised with regard to generalizing the findings. Future studies can use a prospective longitudinal research design and collect data in more cities. Second, the variables were self-reported, and underreporting cannot be ruled out resulting from social desirability bias, Future studies may overcome this limitation through communicating more with the participants and taking feasible and effective measures to gain more trust to strengthen the authenticity of the data. Third, we only considered the comparison of discrimination perception between YMSMs who were in or had been in a marriage and who were not. possibly in future studies, the content of the questionnaire can be improved to create additional category like "Being in a relationship but not marred”.

## Conclusions

Our findings highlight the importance of considering social support and resilience in the effect of sexual minority stigma on mental health among YMSMs. Reducing stigma and giving more social support are effective measures for intervening in the mental health of YMSMs. The chain mediating effect results showed that sexual minority stigma, sexual minority identity, social support, and resilience regulate and influence each other and provide a basis for YMSMs’ mental health regulation. Combined with longitudinal data, this study’s findings could be used to inform interventions targeting mental health and HIV prevention among YMSMs in China.

## Data Availability

The datasets used and analyzed during the current study available from the corresponding author on reasonable request.
